# Graft considerations for successful anterior cruciate ligament reconstruction

**DOI:** 10.1186/s43019-019-0003-5

**Published:** 2019-06-28

**Authors:** Hee-Soo Kyung

**Affiliations:** Department of Orthopedic Surgery, School of Medicine, Kyungpook National University, 130 Dongdeok-ro, Jung-gu, Daegu, 41944 South Korea

For successful anterior cruciate ligament (ACL) reconstruction, several factors, such as preoperative planning, operation technique, and postoperative rehabilitation, have to be considered [1, 2]. Graft choice, fixation, preparation method, maturation, incorporation to host bone, and graft tension should also be considered for good outcome after ACL reconstruction [2]. Several autografts, including the bone-patellar tendon-bone (BTB), hamstring tendon (HT), and quadriceps tendon-bone (QT-B), have been introduced. However, each has its own advantages and disadvantages [3].

The theme of this issue of *Knee Surgery & Related Research* is the preparation method of HT autograft and the evaluation of the graft maturation in ACL reconstruction. Graft preparation for the HT during ACL reconstruction can be performed by several methods, mostly quadruple semitendinosus & gracilis (ST&G), triple ST&G, and quadruple ST [3, 4]. Adequate graft length and diameter are very important for good graft preparation. Adequate graft length is important for early fixation strength in bone tunnel and then for accelerated rehabilitation, so it is usually recommended to be over 7 cm [4]. The graft diameter, which affects the graft re-rupture rate, is usually recommended to be over 7 mm [5, 6].

Dr. Goyal’s report about HT graft preparation is a very interesting article. They compared weave graft and parallel graft preparations to make graft length over 8 cm and graft diameter 7~10 mm. Weave preparation of three-strand ST could be made thicker in diameter, which reduced the gracilis harvesting compared with the parallel graft. Weave graft preparation did not compromise functional outcome and seems to have better graft re-rupture rate 2 years after the operation. So the authors expected preserving the strength in deep flexion and internal rotation of tibia through preserving the gracilis tendon.

Another report about ACL reconstruction, by Dr. Kim, is a systematic review related to evaluation methods of graft maturation on second-look arthroscopy in which 28 studies were analyzed. Graft integrity, tension, and synovial coverage were most frequently evaluated for graft maturation on second-look arthroscopy. Although a few studies reported that the graft tension was significantly correlated with objective stability, second-look findings seem to be less correlated to the clinical outcomes [7–9]. Graft integrity and synovial coverage had no correlation with stability in the included studies [7–10]. There was also no correlation between second-look findings and patient-reported outcomes [7, 9, 11]. Kim et al. concluded that these results may be due to the subjective evaluation of the second-look arthroscopy and the limitations of using evaluation methods that have not yet been validated.

The recent increased use of allograft for ACL reconstruction is due to no donor site morbidity, decreased surgical time, diminished postoperative pain, and good availability of source [12]. However, there were no reports that suggest that allograft may have a better long-term outcome than autograft. Allografts have inherent disadvantages, including a longer and less complete course of incorporation, remodeling, biomechanical inferiority to autograft, and the potential risk of immunogenic reaction and disease transmission [13, 14]. Allograft remodeling is delayed in ACL reconstruction and results in reduced long-term stability and mechanical function compared with autograft ACL reconstruction [13, 14]. Higher long-term failure rates and poorer graft maturation score for allograft were reported compared with those for autograft [15, 16]. The autograft in ACL reconstruction should remain the gold standard, although the allograft is a reasonable alternative.

If adequate length and diameter of autograft can be obtained for ACL reconstruction, autograft with adequate graft fixation and postoperative rehabilitation should be chosen instead of allograft to expect better results. Every effort to enhancing graft maturation and to make a strong graft should be made for a better outcome after ACL reconstruction.

## Abbreviations

ACL: Anterior cruciate ligament
HT: Hamstring tendon
ST&G: Semitendinosus & gracilis
